# Integrating multiple administrative databases to improve the validation and ascertainment of alcohol-related hepatitis

**DOI:** 10.23889/ijpds.v9i5.2705

**Published:** 2024-09-18

**Authors:** Meng Wang, Bing Li, Liam Swain, Abdel Aziz Shaheen

**Affiliations:** 1 Provincial Data Research Services, Alberta Health Services; 2 Department of Community Health Sciences, University of Calgary; 3 Division of Gastroenterology and Hepatology, Department of Medicine, Cumming School of Medicine, University of Calgary

## Objective

This study aims to validate coding algorithms for alcoholic hepatitis (AH) to enhance the precision of case definitions in epidemiologic studies employing administrative data.

## Methods

We used the Inpatient Discharge Abstract Database and Provincial Laboratory & Connect Care databases to identify adult patients affected by AH in Alberta between 2012 and 2022. The AH case definition involved having an International Classification of Disease-10 code K70.1 and fulfillment of at least three laboratory criteria per the National Institute of Alcohol Abuse and Alcoholism (NIAAA): (1) elevated alanine aminotransferase (ALT) or aspartate aminotransferase (AST) >50 IU/L, (2) AST:ALT ratio ≥2, (3) elevated international normalized ratio (INR) ≥1.2, or (4) elevated total bilirubin >30 μmol/L. We compared the diagnostic accuracy of different combinations of these criteria by reviewing a total of 80 randomly selected medical charts (gold standard).

## Results

The proposed algorithm demonstrated a positive predictive value (PPV) of 95% (95% confidence interval [CI]: 75%–100%) when at least three lab criteria were met. However, the estimated PPV decreased to 75% (95% CI: 51%–91%) when only two of the four criteria were met. The estimated PPV was 60% (95% CI: 26%–88%) for cases meeting either elevated INR or elevated total bilirubin, and 50% (95% CI: 19%–81%) for cases meeting either elevated ALT or AST or an increased AST:ALT ratio.

## Conclusion/Implications

In our study, we demonstrated that it is critical to have at least three laboratory criteria to achieve acceptable accuracy of AH case definition.

